# Biliary Ascariasis in the Indian Subcontinent: A Study of 42 Cases

**DOI:** 10.4103/1319-3767.48970

**Published:** 2009-04

**Authors:** Madhumita Mukhopadhyay

**Affiliations:** Department of General Surgery, Calcutta National Medical College and Hospital, Kolkata, West Bengal, India

**Keywords:** Acute upper abdominal pain, biliary ascariasis, ultrasonography

## Abstract

**Background/Aim:**

History of ascariasis is known to stretch back many centuries. One quarter of the world's population is known to be infected by ascariasis. It is endemic in various parts of the Indian subcontinent and the gangetic plain of West Bengal is one of them. We aimed to study the various types of clinical presentations, complications and different diagnostic tools and to assess various options for the management of biliary ascariasis.

**Materials and Methods:**

Forty-two cases of hepatobiliary ascariasis were studied over a period of 3 years. All the patients were adults aged between 20 and 50 years and all but two were admitted with acute upper abdominal pain.

**Results:**

In this study, biliary ascariasis was found to be more common in females, 73.8% (31 patients). The most common presentation was upper abdominal pain in 95.2% of the patients (40 patients). Complications observed were obstructive jaundice in 28.56% (12 patients), cholangitis in 16.7% (seven patients), acute pancreatitis in 2.4% (one patient) and hepatic abscess in 2.4% (one patient). History of worm emesis was present in 38.1% (16 patients). History of previous cholecystectomy was present in 16.7% (seven patients) and endoscopic sphincterotomy in 4.8% (two patients). Ultrasound was the diagnostic tool of choice with 100% results. Conservative management was successful in 83.3% (35 patients). During follow-up, worm reinvasion of the biliary system occurred in 7.1% (three patients).

**Conclusion:**

In endemic countries, ascariasis should be suspected in patients with biliary disease, especially if a cholecystectomy or sphincterotomy has been performed in the past. Most of the patients respond to conservative management.

***Ascaris lumbricoides***, a nematode, is the causative agent of ascariasis. It is the most common helminthic infection in the world. It is distributed throughout the tropics and subtropics. It is prevalent mainly in the developing countries where it usually affects people from the lower socioeconomic groups living in unhygienic conditions.

The adult round worm normally lives in the small intestine. Because they have ‘wanderlust’ and tend to explore ducts and cavities,[1] they often invade the bile or pancreatic ducts. After cholelithiasis, it is the second most common cause of acute biliary symptoms worldwide. Most of the patients in this study presented in the emergency with acute upper abdominal colicky pain. In endemic areas, biliary ascariasis is a frequent diagnosis and should be kept in mind as a cause of acute upper abdominal pain.

## AIMS AND OBJECTIVES

1. To study the various types of clinical presentations of biliary ascariasis and their frequency.
2. To study the various predisposing factors.
3. To study the different types of complications and their frequency.
4. To assess the efficacy of the various diagnostic tools.
5. To assess various treatment options for the management of biliary ascariasis.

## MATERIALS AND METHODS

A total of 42 patients (31 females and 11 males) with biliary ascariasis were studied over a period of 3 years in the Calcutta National Medical College and Hospital, Kolkata, West Bengal, India. All were adults aged between 20 and 50 years belonging to various parts of West Bengal.

Clinical assessment was performed in all the cases. History of passage of worms in the stool or vomitus and recurrent abdominal pain, with or without jaundice, was taken in each case. Previous history of surgery or endoscopy to the gastrointestinal tract was noted. Complete blood cell count, liver function test, serum amylase, X-ray of the chest and abdomen and ultrasound of the abdomen was performed in all the patients at the time of admission and repeated when required. The mainstay of the diagnosis was ultrasound of the abdomen.

All the patients were initially managed conservatively with IV fluids, IV antibiotics and IV antispasmodics. All the patients were dewormed with a single dose of 400 mg of albendazole. Endoscopic or surgical invention was carried out when conservative treatment failed. Serial ultrasonography was performed to check for recurrence during follow-up. The patients were dewormed at 6-monthly intervals.

## RESULTS

In this study, biliary ascariasis was found to be more common in females (73.8%). The most common presentation was upper abdominal pain in 95.2% of the patients. The clinical symptoms and signs are given in Table 1. The white blood cell count was moderately raised, with eosinophilia in most patients. Alkaline phosphatase was raised in 19 patients and serum amylase was raised in one patient.

**Table 1:** Clinical presentations

Symptoms	No. of patients	Percentage
Pain (right) upper quadrant of the abdomen (most common)	40	95.2
Nausea and vomiting	32	76.2
Vomiting of worms	16	38.1
Worms in stool	23	54.8
Fever	7	16.7
**Signs**		
Right upper quadrant tenderness	30	71.4
Hepatomegaly (tender)	8	19.1
Jaundice	12	28.6
Palpable GB lump	2	4.8

Eleven patients had previous history of surgery of the gastrointestinal tract. The different types of surgeries are given in Table 2. Complications occurred in 21 patients. The various types of complications are given in Table 3.

**Table 2:** Previous surgery of the gastrointestinal tract

**Type of surgery**	**No. of patients**	**Percentage**
Cholecystectomy	7	16.7
Endoscopic sphincterotomy	2	4.8
Gastrojejunostomy	1	2.4
Choledochoduodenostomy	1	2.4

**Table 3:** Complications

**Complications**	**No. of patients**	**Percentage**
Obstructive jaundice	12	28.6
Cholangitis	7	16.7
Pancreatitis	1	2.4
Hepatic abscess	1	2.4

About 83% of the patients responded to conservative therapy. Most of the patients responded to this treatment in 4–5 days. In some patients, the above treatment had to be continued up to 10 days. Patients were monitored by serial ultrasound to know the status of the worms in the biliary tree. Endoscopic extraction was performed in five cases. The indications were retention of dead worms in the common bile duct with obstructive jaundice. Cholecystectomy with common bile duct exploration was carried out in two patients with coexistent choledocholithiasis where endoscopic retrograde cholangiopancreatography failed to remove the stones. Among the two patients who underwent surgical exploration, one patient had a small, partially ruptured liver abscess close to the gall bladder fossa. When it was drained, a small fragment of a dead round worm was found in the cavity. Common bile duct exploration revealed another dead round worm along with choledocholithiasis.

Patients were followed-up for 6 months with serial ultrasonography. Worm reinvasion of the biliary tract occurred in three patients (7.1%). Of the three patients, one patient had a prior history of endoscopic sphincterotomy and another had a prior history of choledochoduodenostomy. Reinvasion was successfully managed by conservative therapy.

## DISCUSSION

Biliary ascariasis is commonly reported from highly endemic regions like the Fareast, Indian subcontinent, Latin America, parts of the Middle East and Africa. In humans, the usual habitat of ***A. lumbricoides*** is the small intestine. When the worm load is high, which may go up to a 1000 worms, the worms tend to migrate away from the usual site or habitat.

**Figure 1:**
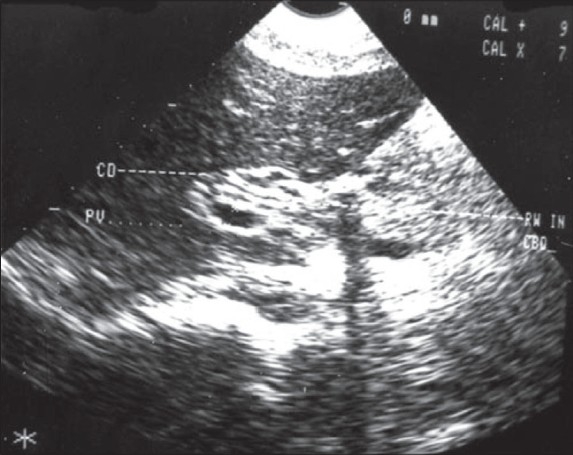
Ascaris in the common bile duct

**Figure 2:**
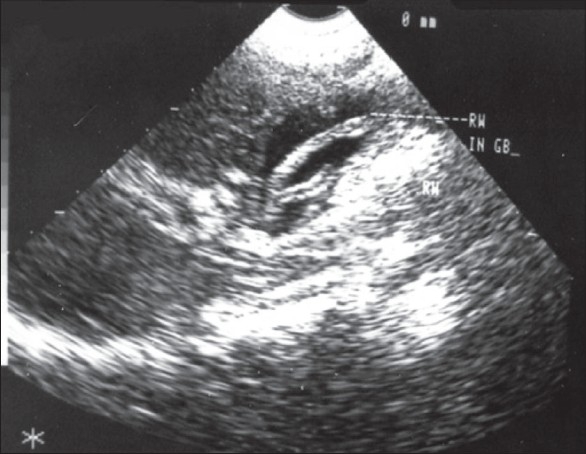
Ascaris in the gall bladder

**Figure 3:**
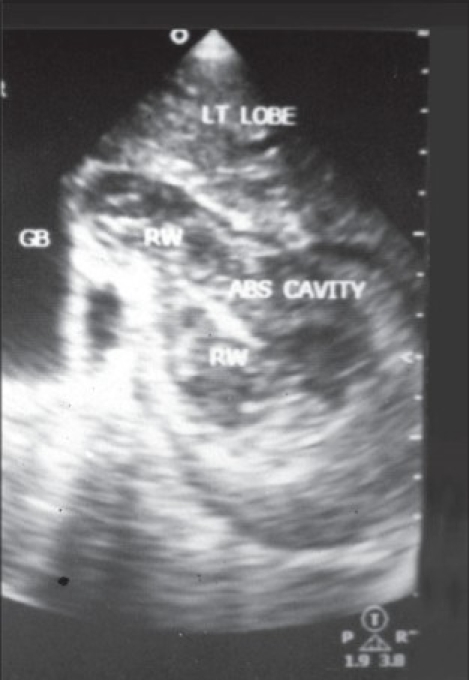
Ascaris in the hepatic abscess cavity

Symptoms of biliary colic occur when the worm migrates across the papilla. If the worm remains in the bile duct [Figure 1] and gall bladder [Figure 2], acute and chronic complications can occur, like cholangitis, strictures, calculi, cholecystitis and pancreatitis.[3] Some worms may travel up and colonize in the liver parenchyma forming liver abscess[4] [Figure 3].

Women are more commonly affected than men. Recurrent worm invasion of the ducts has also been frequently observed in endemic regions. Khuroo ***et al***, in their study, reported a similar observation.[5] Seventy-six of the 500 patients studied had worm reinvasion. Predisposing factors for recurrent worm invasion include previous cholecystectomy or sphincterotomy or even prolonged fasting, as reported by a recent study.[6]

Previous surgery on the biliary tract predisposes to biliary ascariasis. Cases have been reported after sphincterotomy and Roux-en-Y hepaticojejunostomy.[5–8] Some studies have shown that almost 30% of the patients with biliary ascariasis have a prior history of cholecystectomy.[9] Following cholecystectomy, there is a dilatation of the common bile duct as well as a rise in cholecystokinin, which in turn leads to a relaxation of the sphincter of oddi.

The diagnosis of biliary ascariasis usually depends on the demonstration of worms in the biliary tract by different imaging techniques. Sonography has been shown to have a high diagnostic accuracy as a noninvasive procedure in the diagnosis of biliary ascariasis.[10] Various appearances of round worms in the biliary tract and gall bladder have been described.[10,11] They are as follows:

1. Inner tube sign – The round worm may be seen as a thick echogenic stripe with a central anechoic tube (gastrointestinal tract of the worms) in the gall bladder or common bile duct.
2. Stripe sign – Thin nonshadowing stripe without an inner tube within the gall bladder or common bile duct.
3. Spaghettli sign – Overlapping longitudinal interfaces in the main bile duct due to coiling of a single worm or several worms in the common bile duct.

In addition, real time sonography may demonstrate mobility of the worms within the gall bladder and biliary passages thus equivocally establishing the diagnosis. Ultrasonography is also helpful in monitoring the exit of the worms from the biliary tract.

Endoscopic retrograde cholangio pancreatography (ERCP) usually shows the worm as a long filling defect. Successful extraction of the worm from the common bile duct via endoscope has been reported in the literature.[12–14] But, the use of ERCP must be balanced against potential complications of the procedure. Moreover, sphincterotomy performed during ERCP for worm extraction predisposes to recurrent worm infestation. Because this disease is more rampant in the poorer tropical countries of the world, the expense of an ERCP adds significantly to the overall cost of treatment. This holds true for India. Therefore, ERCP as a therapeutic intervention should be considered if a patient fails to respond to conservative treatment or the worm persists (serial sonograms) or has died within the pancreaticobiliary tree.[15] Presence of coexistent strictures or stones within the ducts is also an indication.

More than 95% of the patients with uncomplicated biliary ascariasis will respond to conservative management, the worms returning spontaneously to the intestine.[3,4] In a study from Kashmir, conservative management was successful in 88% of the patients.[16]

## CONCLUSION

In endemic areas, biliary ascariasis is a frequent diagnosis in patients presenting with symptoms of biliary colic. Most of the patients of biliary ascariasis respond to conservative treatment. Ultrasonography of the abdomen has been advocated as a quick, safe, noninvasive and relatively inexpensive modality with a high diagnostic accuracy for suspected biliary ascariasis.
